# Bacterial membrane vesicles in the pathogenesis and treatment of inflammatory bowel disease

**DOI:** 10.1080/19490976.2024.2341670

**Published:** 2024-04-26

**Authors:** Chinasa Valerie Olovo, Dickson Kofi Wiredu Ocansey, Ying Ji, Xinxiang Huang, Min Xu

**Affiliations:** a Department of Gastroenterology, Affiliated Hospital of Jiangsu University, Zhenjiang, Jiangsu, China; b Department of Biochemistry and Molecular Biology, School of Medicine, Jiangsu University, Zhenjiang, Jiangsu, China; c Institute of Digestive Diseases, Jiangsu University, Zhenjiang, Jiangsu, China; d Department of Microbiology, Faculty of Biological Sciences, University of Nigeria, Nsukka, Nigeria; e Key Laboratory of Medical Science and Laboratory Medicine of Jiangsu Province, School of Medicine, Jiangsu University, Zhenjiang, P.R. China; f Department of Medical Laboratory Science, School of Allied Health Sciences, College of Health and Allied Sciences, University of Cape Coast, Cape Coast, Ghana

**Keywords:** Inflammatory bowel disease, bacterial membrane vesicles, onset, progression, diagnosis, therapy

## Abstract

Inflammatory bowel disease (IBD) is a chronic and debilitating condition of relapsing and remitting inflammation in the gastrointestinal tract. Conventional therapeutic approaches for IBD have shown limited efficacy and detrimental side effects, leading to the quest for novel and effective treatment options for the disease. Bacterial membrane vesicles (MVs) are nanosized lipid particles secreted by lysis or blebbing processes from both Gram-negative and Gram-positive bacteria. These vesicles, known to carry bioactive components, are facsimiles of the parent bacterium and have been implicated in the onset and progression, as well as in the amelioration of IBD. This review discusses the overview of MVs and their impact in the pathogenesis, diagnosis, and treatment of IBD. We further discuss the technical challenges facing this research area and possible research questions addressing these challenges. We summarize recent advances in the diverse relationship between IBD and MVs, and the application of this knowledge as a viable and potent therapeutic strategy for IBD.

## 1. Introduction

Inflammatory bowel disease (IBD) is a chronic intestinal inflammation and mucosal immune-associated illness that involves dysbiosis of the intestinal microenvironment ,^1–3^ The two main subtypes of IBD, ulcerative colitis (UC) and Crohn’s disease (CD), are typified by debilitating and chronic relapsing and remitting inflammation in the colon and gastrointestinal tract (GIT).^4^ Although the cause of IBD is still unclear, it has been described as multifactorial, involving the combination and interplay of genetic susceptibility, immune dysregulation, microbial factors, and environmental triggers.^5,6^ While some conventional medications exist for the treatment of IBD with 5-aminosalicylates (5-ASAs), corticosteroids, and immunosuppressive agents as mainstay drugs,^7^ they have, however, shown limited efficacy and detrimental side effects leading to the quest for new and effective treatment options for the disease.

The relationship between IBD and the gut microbiota has been well established by many studies,^8–11^ The gut microbiota is vital in maintaining intestinal homeostasis and function, integrity of the epithelial barrier, and health and disease. The biodiversity and number of gut microbiota can be shaped by a variety of factors ranging from exposure to antibiotics, exogenous enzymes, prebiotics, probiotics, fecal microbiota transplantation, diet, and a host of diseases ,^12–14^ These factors can in turn, cause a disruption of the microbiota, leading to an abnormally composed microbiota referred to as “dysbiosis” as opposed to “eubiosis.” A large number of human disease conditions, including but not limited to diabetes type II, allergies, colorectal cancer, obesity, cardiovascular diseases, and IBD have been linked to an altered composition of the microbiota.^13,15,16^ Accumulating evidence indicates that bacteria release vesicles that facilitate the actions of the microbiota by transferring and delivering effector chemicals into host cells that modulate host signaling pathways and cell activities. Thus, vesicles secreted by the gut microbiota could have a significant impact on the health and illness of the host.^17^

Bacterial membrane vesicles (MVs) are nano-sized lipid-bilayered vesicular structures composed of various immunostimulatory components.^18^ The sizes range from 20 to 400 nm in diameter for Gram-positive bacteria^19^ and 20 to 250 nm for Gram-negative bacteria.^20,21^ MVs, which were originally discovered to be generated through controlled blebbing of the outer membrane of Gram-negative bacteria, and referred to as outer membrane vesicles (OMVs),^22^ were initially disregarded as bacterial artifacts. Early investigations, conducted in the 1960s depicted OMVs being released from the outer membrane of various Gram-negative bacteria through electron microscopy. Nonetheless, it was not until the detection of OMVs in the spinal fluid of meningococcal patients that curiosity arose in comprehending OMV generation, their roles within the host, and their advantageous attributes for bacteria. ^23–25^ Relative to Gram-negative bacteria, Gram-positive bacteria lack an outer membrane but instead contain a thick peptidoglycan cell wall, resulting in the initial disinterest in MVs research for the bacteria. Although vesicle-like blebbing structures were reported on the surface of *Bacillus* spp., it was not until 2009 that the first characterization of MVs from the Gram-positive bacteria, *Staphylococcus aureus* was made with the aid of mass spectrometry.^19^ MVs from both Gram-negative and Gram-positive bacteria perform functions that influence diverse biological processes, which can either be between bacteria – bacteria or bacteria – host cells.^20,22,26^ In recent times, studies have revealed that MVs are implicated in the onset and progression, as well as in the treatment of IBD.^27–31^

In this review, we introduce MVs and give a general overview of their biogenesis, composition, and functions. We focus on the potential involvement of MVs in the onset and progression of IBD, as well as in the diagnosis and treatment of the disease . We discuss specific roles played by MVs in their interactions with the gut microbiota, intestinal epithelial cells (IECs), and immune system, that could trigger and/or exacerbate inflammation. We reveal the potential of MVs as diagnostic biomarkers of IBD and therapeutic agents, either as the active ingredient or as a drug carrier. The present review also explores the possibility of harnessing MVs for IBD vaccines and in genetic engineering to broaden and enhance therapeutic outcomes. Additionally, we present a critical analysis of the present challenges facing MVs-IBD research and propose future research paths that could be explored to tackle these challenges.

## 2. Overview of MVs

**Figure 1.**
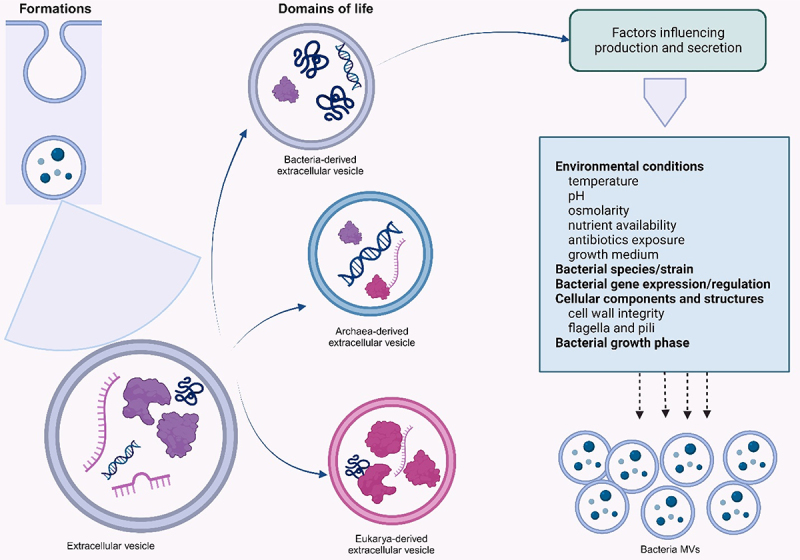
Formation of MVs from the three domains of life and factors that influence their secretion.

Extracellular vesicles (EVs) are nano-sized particles surrounded by a lipid bilayer. They are excreted by a cell to the extracellular environment. Cells from the three domains of life, Archaea, Bacteria, and Eukarya, produce EVs, and their release follows a common and possibly conserved process within various species,^32–34^ (Figure 1). While the EVs from Gram-negative bacteria are called OMVs, vesicles from Gram-positive bacteria are known as membrane vesicles (MVs) or cytoplasmic membrane vesicles (CMVs) due to lack of an outer membrane in the bacteria and their mode of formation.^22,35^

The three life domains are Eukarya, Archaea, and Bacteria.

MVs production and secretion are majorly influenced by the expression and regulation of the parent bacterial genes, producing bacterial species, bacterial growth phase, cellular components and structures, and environmental conditions including the bacteria’s growth conditions.^36,37^

### 2.1 Biogenesis

Several studies have described two key routes for the generation of MVs in Gram-negative bacteria: blebbing of the outer membrane and endolysin-triggered cell lysis.^4,22^ Blebbing of the outer membrane in Gram-negative bacteria is reported to occur as a result of a disturbance in the cell envelope due to intercalating of the hydrophobic molecules into the outer membrane or from the unbalanced biosynthesis of the cell membrane.^4,22^ Three mechanisms that involve membrane blebbing have been described and these include the reduced cross‐linking between the outer membrane and the underlying peptidoglycan,^38,39^ the accumulation of peptidoglycan fragments or misfolded proteins in the periplasmic space,^39^ and vesicles derived from bacterium flagellar-sheaths upon rotation of the flagella.^39^

Various disturbances, such as an imbalance of peptidoglycan biosynthesis, could consequently lead to disruption of crosslinking between peptidoglycan and the outer membrane, causing dissociation of the outer membrane from the peptidoglycan layer.^40^ Studies have shown that certain bacteria, such as *Escherichia coli*, *Vibrio cholerae*, and *Salmonella* spp. mutants deficient in OmpA (an outer membrane porin bearing a periplasmic binding site for diaminopimelic acid, which is a component of peptidoglycan), exhibit increased release of their MVs compared to the wild-type strains.^20,40,41^ The hypervesiculating nlpI mutant has around 40% less lipoprotein crosslinked to peptidoglycan than wild-type *E*. *coli*.^42^ Nlpl is an outer membrane lipoprotein involved in cell division and in the regulation of the activity of Spr (MepS), a peptidoglycan endopeptidase that breaks down peptide crosslinks in peptidoglycan.^20,43^ Hence, it is proposed that the altered balance of peptidoglycan synthesis and breakdown in nlpI mutants inhibits the development of appropriate crosslinks between peptidoglycan and lipoprotein and, consequently, increases the release of MVs.^20^ The buildup of peptidoglycan fragments or misfolded proteins in the periplasmic space is the second mechanism that results in the generation of MVs by blebbing, as displayed by *E*. *coli* and *P*. *aeruginosa*.^39,44,45^ Mutants of *Porphyromonas gingivalis* deficient in autolysin demonstrated an increase in MVs production, and this emanated from the inability of the bacterium to breakdown periplasmic peptidoglycan fragments that accumulated in the periplasm due to the lack of autolysins in *P*. *gingivalis*.^46,47^ Bacteria with mutations in their envelope stress pathways are incapable of protein degradation, and this can result in the accumulation of misfolded proteins, which exert pressure on the membrane of these bacteria, ultimately leading to increased MV secretion. As reported by McBroom and Kuehn in their study, higher growth temperatures cause an increase in the vesiculation of *E*. *coli*.^48^ Lastly, the assembly of bacteria flagella, particularly sheathed flagella, also occasion membrane blebbing of vesicles. The flagella are surrounded by a sheath derived from the outer membrane and, upon rotation, release MVs, and this phenomenon has been reported to occur in members of *Vibrio* spp.^49^

Endolysin-triggered cell lysis, on the other hand, involves vesicle formation routes based on the enzymatic actions of endolysins, typically employed by double-stranded DNA phages that utilize these peptidoglycan-hydrolyzing enzymes in the lysis of their hosts for the release of their progeny. Consequently, the cells round up and explode releasing fragments of shattered membrane that round up and self-assemble into E-type MVs.^50^ This type of MV biogenesis has been observed in *P. aeruginosa*.^51^ MVs that arise from Gram-negative bacteria’s explosive cell lysis carry endolysins and have the ability to lyse other cells,^52^ generating new MVs.^34^

In Gram-positive bacteria, MVs are released by a process known as “bubbling cell death,” which is somewhat similar to explosive cell death in Gram-negative bacteria. This process of MV biogenesis has been observed in *Bacillus subtilis*,^53^ *Lacticaseibacillus casei*,^54^ and in other Gram-positive bacteria as well. ^55–57^ A sub-population of cells of *B. subtilis* express a prophage-encoded endolysin causing holes in the peptidoglycan cell wall. As a result, materials of the cytoplasmic membrane bulges into the extracellular area and is released as MVs.^53^ Endolysins secreted from dying *B. subtilis* cells have been demonstrated to cause MV formation in nearby cells by hydrolyzing the cell wall from the outside. In *S. aureus*, a type of blebbing mechanism has been proposed for MV biogenesis. It involves the disruption of the cytoplasmic membrane by amphipathic, α-helical, phenol-soluble modulins. Subsequently, autolysins, which weaken the crosslinking of the peptidoglycan, modulate MV release through the cell wall.^58^ Peptidoglycan-hydrolyzing enzymes or β-lactam antibiotics^57,59–61^ also promote the weakening of the cell envelope, resulting in MV formation in some Gram-positive bacteria.

### 2.2. Composition

**Figure 2.**
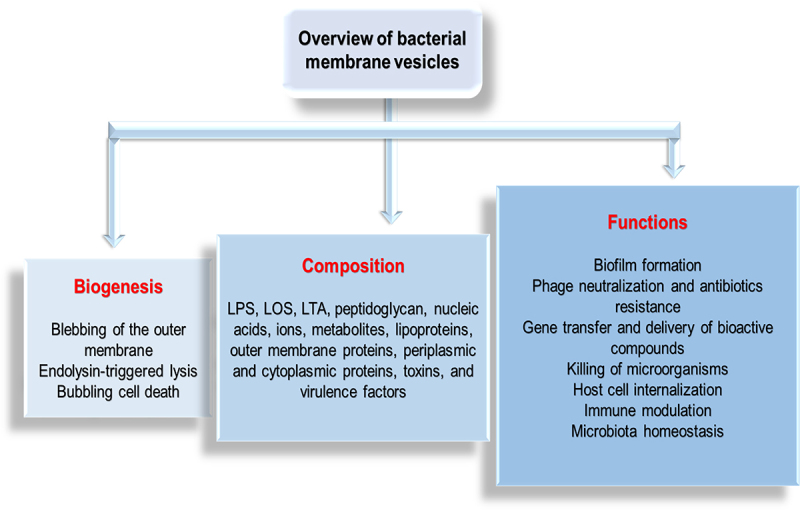
Overview of bacterial membrane vesicles.

The cargo molecules in MVs are diverse due to variations in the parent bacteria, the biogenesis route, and other environmental factors. This diversity facilitates the roles MVs play in bacteria-bacteria and bacteria-host interactions. The MVs of Gram-negative bacteria have been reported to contain numerous parental components, including enzymes, lipopolysaccharides (LPS), lipooligosaccharides (LOS), proteins, nucleic acids, phospholipids, outer membrane proteins (OMPs), periplasmic and cytoplasmic proteins, cell wall components, ions, metabolites, and signaling molecules.^20,62^ Unlike MVs from Gram-negative bacteria, Gram-positive bacteria MVs lack LPS and periplasmic components, while other cargo molecules, including peptidoglycan, lipids, lipoproteins, proteins, and nucleic acids, remain the same.^19^ Lipoteichoic acid (LTA), however, is an exclusive component of the Gram-positive bacteria MVs.^19^ Pathogens, toxins, and virulent factors are also part of the MVs’ composite of both bacteria Gram-types^17^ (Figure 2). The differences between MVs produced by Gram-positive and Gram-negative bacteria are summarized in Table 1.

This overview centers on general knowledge regarding the biogenesis, composition, and functions of bacterial MVs.

**Table 1.** Differences between MVs produced by Gram-negative bacteria and Gram-positive bacteria.

S/N	Characteristics	Gram-negative bacteria	Gram-positive bacteria
1.	Composition	Relative to MVs from Gram-positive bacteria, MVs from Gram-negative bacteria contain LPS, LOS, peptidoglycan (10–20%), outer membrane, and periplasmic proteins.^20,35,47,57,63^	Unique to MVs from Gram-positive bacteria are peptidoglycan (>50%) and LTA.^19,26,35,63,64^
2.	Size	MVs generally have a smaller size, ranging from 20 to 250 nm in diameter.^21^	The diameter of MVs is larger with ranges of 20–400 nm in diameter.^19^
3.	Delivery of virulence factors	Enzymes involved are phospholipase C, esterase lipase, alkaline phosphatase, and serine protease and the toxins are adenylate cyclase toxin, cholera toxin, and cytolethal distending toxin.^65^	Enzymes include IgG-binding protein Sbl, protective antigen, lethal factor, edema toxin, and anthrolysin.^65^
4.	Biogenesis	MVs are formed by two major pathways: membrane blebbing (involving disruption of crosslinking between peptidoglycan and the outer membrane, accumulation of peptidoglycan fragments or misfolded proteins in the periplasmic space, assembly of sheathed flagella) and explosive cell lysis.^22,39^	MVs are formed by the activity of peptidoglycan-hydrolyzing enzymes such as autolysins, endolysins, and the β-lactam antibiotics.^53,58^
5.	Host cell modulation	VacA toxin, cytolysin A, α-hemolysin, Cif, flagellin, shigatoxin, and heat-labile enterotoxin^65^ in MVs are involved.	α-hemolysin: proteolysin, β2 toxin, and superantigens: SEQ, SSaA1, and SSaA2^65^ in MVs of Gram-positive bacteria carry out this activity.
6.	Killing competing bacteria	To carry out this activity, murein hydrolase (Mlt, Slt), endopeptidase L5, and peptidoglycan hydrolase present in MVs are employed.^65^	N-acetylmuramoyl-L-alanine amindase in MVs are employed.^65^
7.	Bacteria adhesion and invasion	The presence of adhesin, invasion, and OmpA^65^ in MVs facilitate this activity.	The presence of plasma-binding proteins and staphopain A in MVs^65^ enable this activity.
8.	Antibiotic resistance	β-lactamase, enzyme L5, and multidrug efflux protein (*Mtr*, *Mex*, *TolC*)^65^ are present in MVs.	β-lactamase including penicillin-binding proteins: PBP1, PBP2, PBP3, and PBP4^65^ are found in MVs.
9.	Coagulation	Thrombomodulin, E-selectin, and P-selectin^65^ in MVs of Gram-negative bacteria carry out this function.	Von Willebrand factor-binding protein, staphylocoagulase precursor, and fibronectin-binding protein^65^ present in MVs are implicated.
10.	Source	Vesicles are known as OMVs since they are formed from the outer membrane.^20,22^	Vesicles are known as MVs or CMVs since they originate from the cytoplasmic membrane^19,22^

LTA - lipoteichoic acid, LPS -Lipopolysaccharides, LOS – Lipooligosacchaarides, OmpA – outer membrane protein A, OMV – outer membrane vesicle, CMV – cytoplasmic membrane vesicle.

Many factors such as bacteria growth stage, conditions of the growth medium, and other environmental factors affect the generation and content of MVs. For instance, The culture of *Vibrio vulnificus* under optimized conditions of 37°C in an enriched medium of 2 × Luria Bertani in the presence of EDTA significantly increased the production of their MVs by about 70%.^66^ Again, MVs derived from *P. gingivalis* at different growth stages not only determined the MV yield but also the protein content and periodontal pathogenicity of these MVs. MVs were extracted in the pre-log, late-log, and stationary growth phases of the bacteria, and it was reported that significantly increased yield, protein composition, and pathogenicity were associated with MVs from the stationary phase of growth.^67^

The content of MVs has been analyzed using various methods including bicinchoninic acid (BCA) assay, Sodium Dodecyl Sulfate Polyacrylamide Gel Electrophoresis (SDS/PAGE), Western blotting, enzyme-linked immunosorbent assay (ELISA), mass spectrometry (MS), and colorimetric assays.^68,69^ While the total protein concentration of MVs is quantitatively determined by the BCA assay, SDS/PAGE is a qualitative determination of the total protein content of MVs on a polyacrylamide gel. The presence of a target protein is determined by ELISA and Western blotting. The mass-charge-to-charge ratio (m/z) of gaseous samples can be measured and analyzed in a vacuum environment using the MS technique. With the aid of this high-throughput proteomic analysis, thousands of proteins have been detected and this serves to reveal substantial evidence that supports the biogenesis and functions of MVs.^68,69^ Colorimetric-based assays are employed to ascertain the quantity of LPS present in the MVs. Some examples are the KDO (2-keto-3-deoxyoctonate) assay, (KDO is an essential sugar component of LPS) and Limulus Amebocyte Lysate (LAL) assay.^69–71^ Protein assays, including BCA, Bradford, Lowry, or Qubit assays are the most extensively used methods for quantifying MVs for functional assays. MV protein content, however, can be considerably altered by factors such as bacteria growth stage,^67,72–74^ MV size,^75^ culture conditions,^76–78^ bacterial strains,^79^ and MVs isolation method,^73,80,81^ indicating that MV protein concentration and the quantity of MVs may not be directly correlated.^82^ This reveals that the best method(s) to administer MVs for functional assay purposes need to be determined in order to increase the level of objectivity obtainable in comparative studies of MVs.

### 2.3. Functions

**Figure 3.**
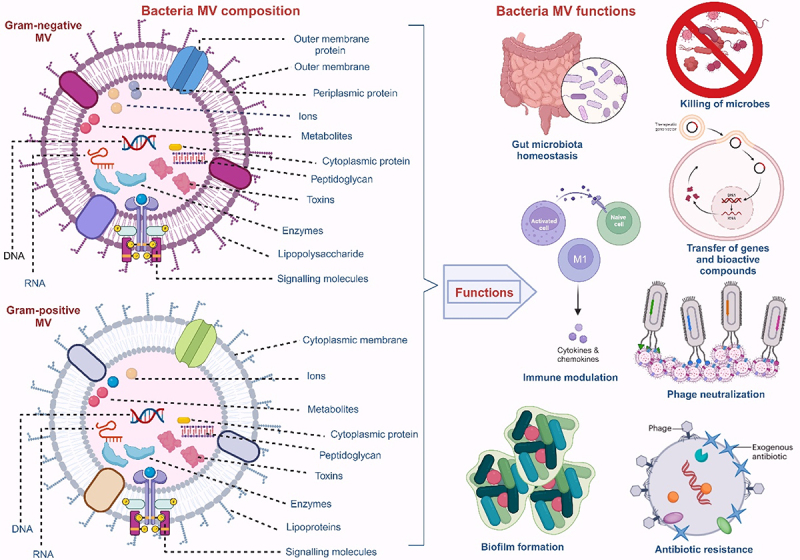
Composition and functions of MVs from Gram-positive and Gram-negative bacteria.

MVs perform important functions (determined by the MV’s structure and composition which are dependent on its biogenesis route), leading to their diverse roles in bacteria and their hosts. These vesicles perform functions that influence diverse biological processes, and which can either be between bacteria – bacteria or bacteria – host cells.^20,22,26^ These functions, which include biofilm formation, gene transfer, antibiotics and phage neutralization, host cell internalization, disease progression, immune modulation, and microbiota homeostasis^68^ (Figure 3) are briefly described below:

The cargos of MVs from Gram-negative bacteria differ slightly from that of Gram-positive. Represented functions include biofilm formation, antibiotics resistance, phage neutralization, immune modulation, gene transfer, the killing of microorganisms, and gut microbiota homeostasis.

#### 2.3.1. Biofilm formation

MVs in microbial communities are known to be key players in biofilm formation by enhancing the stability of the biofilm matrix, and in the facilitation of bacterial colonization due to their ease of spread on biofilm surfaces.^33^ Various reports reveal that hydrophobic quorum-sensing molecules that coordinate bacterial growth and behavior, depend on the population density, and are secreted into MVs. Studies have also revealed that MVs are essential components of the biofilm matrix, usually composed of lipids, proteins, nucleic acids, and polysaccharides.^83^ MVs, thus, transport the necessary molecules that promote biofilm formation. Various studies have elucidated the vital role of extracellular genomic DNA (eDNA) in the onset and stabilization of biofilms. The presence of eDNA in the MVs of *S. aureus*,^84^ *Acinetobacter baumannii*, *Francisella* spp,^85^ and *P. aeruginosa*^86^ promotes biofilm formation.^20,39^ Some of these pathogens have been implicated in various nosocomial infections and burn wounds, with an increased incidence of chronic infections due to the formation of biofilms. ^84,86^ The implications of MVs in biofilm formation and IBD progression are discussed in the next section.

#### 2.3.2. Phages neutralization and antibiotics resistance

**Figure 4.**
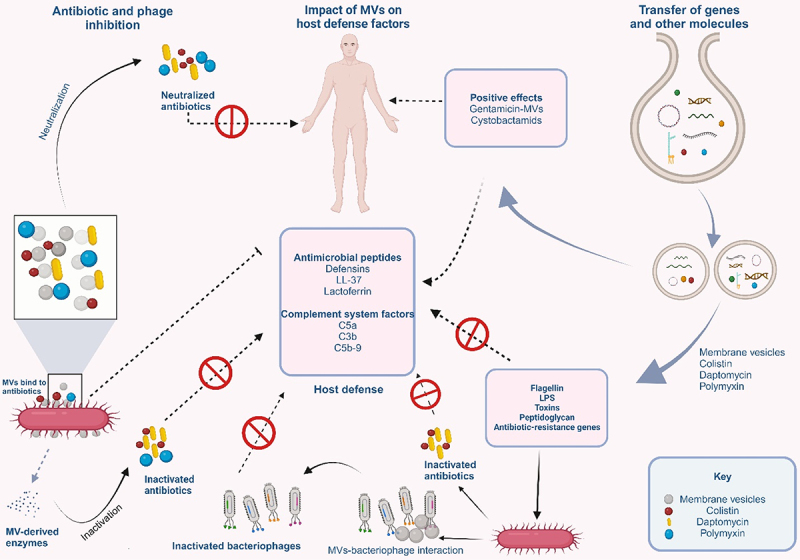
MVs in phage neutralization, antibiotics resistance, gene transfer, and delivery of bioactive compounds.

Agents that bind to bacterial membranes will be adsorbed to MVs. As a result, MVs neutralize antibiotics such as colistin, daptomycin, and polymyxin that target the bacterial membrane^87^ as observed in MVs of *E. coli*.^87,88^ MVs are also known to release enzymes that confer antibiotic resistance to the parent bacteria and other susceptible bacteria in the microbial community^17,89^ (Figure 4). For instance, *S. aureus* and *Moraxella catarrhalis* carry biologically active β-lactamase in their MVs.^89,90^ MVs can also provide antibiotic protection to both the producer strain and other bacterial populations in a given environment^88^ and offer protection against host-defense factors such as antimicrobial peptides from mammalian tissue and complement system factors of the blood.^57^

MVs on the surface of their parent bacteria can neutralize phages by binding to them. They can also inactivate antibiotics by the same mechanism or by releasing enzymes that confer resistance to the parent bacteria. MVs are also involved in the transfer of antibiotic-resistance genes and other virulence factors to different bacteria species. These will in turn inhibit the actions of host defense factors, preventing the elimination of the bacterial pathogens from the system. They can also mediate the transfer of bioactive molecules that can aid host defense factors in the elimination of harmful pathogens.

Additionally, MVs serve to prevent adsorption of phages onto bacteria. This is because the attachment of MVs onto the surface of the producer strain occupies the phage receptors, thereby preventing their binding onto the bacterial cell surface. The phages are then made to bind on the surface of the MVs through the phage receptor proteins on the surfaces of the MVs^91,92^ (Figure 4). While MVs from *E. coli* were reported to neutralize T4 phage, those from *V. cholerae* neutralize ICP1, CIP2, and ICP3 phages.^87,93^

In summary, MVs sequester phages and antibiotics greatly reducing their availability so that they have no direct interaction with the parent bacteria. These observations show the involvement of MVs in the occurrence of antibiotic-resistant bacteria strains.^94^

#### 2.3.3. Gene transfer and delivery of bioactive compounds

Until recently, conjugation, transformation, and transduction were the three major gene transfer mechanisms. However, the discovery of MVs defined a new pathway for gene transfer. The genetic material of up to 370 kb has been discovered in the MVs of Gram-positive, Gram-negative, and archaeal microorganisms. All genetic materials, including chromosomal and plasmid-derived DNA, and RNA variants, have been found in MVs^95,96^ (Figure 4). The interesting study of Carvalho and collaborators demonstrated that engineered MVs from *Bacteroides thetaiotamicron* (*Bt*-MVs) packaged and expressed both *Salmonella enterica* serovar Typhimurium-derived vaccine antigens and influenza A virus (IAV)-derived vaccine antigens within or on the outer membrane of *Bt*-MVs.^97^ These antigens were shown to possess the ability to trigger antibody and antigen-specific immune responses in both mucosal tissues and systemically. This means that MVs can serve as vehicles in the delivery of genetic materials for novel biotechnological applications. Engineered MVs are being developed as new vaccines and adjuvants or as specialized drug delivery vehicles for the treatment of such diseases as cancer.^98,99^

#### 2.3.4. Killing of microorganisms

MVs have the ability to interact with bacteria and other organisms, including eukaryotes and plants.^68,100^ Certain bacterial strains belonging to *Pseudomonas*, *Enterobacter*, *Klebsiella*, and *Citrobacter* genera have been reported to secrete toxin-carrying MVs that can kill other bacteria in a competitive environment.^101^ Besides the killing of other bacteria, some bioactive compounds and lytic enzymes present in MVs can also kill fungi. This can be observed in the MVs of members of the genera *Lysobacter* and *Myxococcus* that lyse and feed on microorganisms. These MVs contain abundant hydrolytic enzymes, which they use to attack their prey. An example is the lytic protease L5 in *Lysobacter* spp. XL1.^57,102^

#### 2.3.5. Host cell internalization

**Figure 5.**
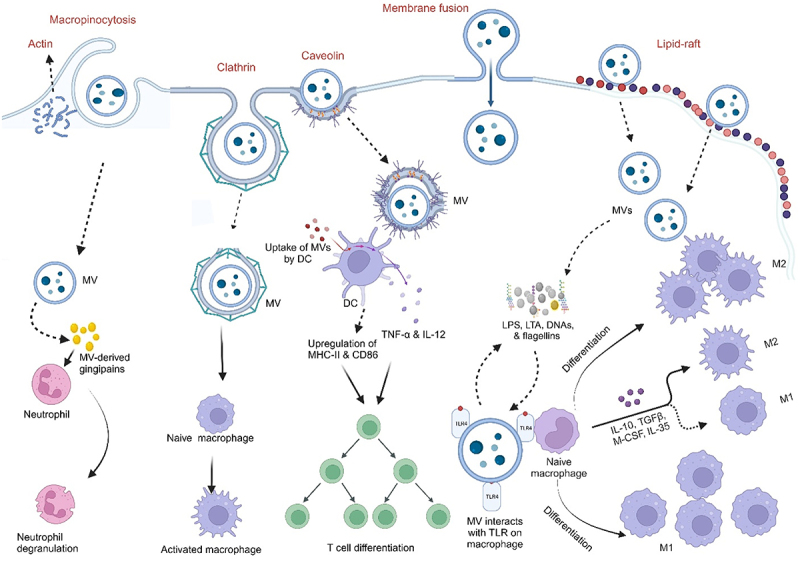
Internalization of membrane vesicles into host cells and modulation of the immune system.

Uptake of MVs by host cells and delivery of their cargo into host cells must occur for a successful interaction between host cells and MVs. For instance, the internalization of LPS-containing MVs of *E. coli* BL21 by human intestinal epithelial cells resulted in the downregulation of E-cadherin expression, and intestinal barrier dysfunction further exacerbating inflammation. Uptake of MVs by non-phagocytic cells has been proposed to occur through five mechanisms, which are macropinocytosis, clathrin-mediated endocytosis, lipid raft-mediated endocytosis, caveolin-mediated endocytosis, and direct membrane fusion^103,104^ (Figure 5). Moreover, the mechanism by which MVs enter the host cells depends on the size and cargo of the MVs.^47^

MVs are internalized by epithelial cells via macropinocytosis (dependent on actin), clathrin-mediated/caveolin-mediated endocytosis, membrane fusion, and lipid-raft. MVs interact with various immune cells upon internalization to elicit an immune response. MVs of *P*. *gingivalis* containing gingipains selectively coat, activate, and consequently degranulate neutrophils to ensure the survival of the parent bacterium. MVs can activate naïve macrophages via interactions of their MAMPS with PRR present in macrophages. Interactions of MVs-derived LPS, LTA, DNAs, and flagellins, with TLRs of macrophages can polarize them to either M1 or M2 phenotype (depending on the producing bacteria, among other factors), inducing the expression of anti-/pro-inflammatory cytokines. DCs activate the expression of cytokines (TNF-α and IL-12) and specific surface molecules (CD86 and MHC-II molecules) that promote differentiation of T-cells to specific functional subsets immediately upon internalization of bacterial membrane vesicles. MAMPS – Microbe-associated molecular patterns; PRR – Pattern recognition receptors, LTA – Lipoteichoic acid, DNA – Deoxyribonucleic acid, TLRs – Toll-like receptors.

Macropinocytosis is channel utilized by viruses, which are similar in size to MVs, and is proposed to be a possible uptake mechanism of MVs by host cells.^105^ Macropinocytosis, which is dependent on actin, involves the formation of large, ruffled protrusions from the cell membrane that permit the sampling and internalization of extracellular medium.^104,106^ The formation of clathrin-coated pits of up to 200 nm in diameter is indicative of clathrin-mediated endocytosis (CME). Here, ligand binding to cell surface receptors can initiate internalization, and dynamin is also needed for the budding off of the vesicle. Unlike macropinocytosis, CME is a well-defined mechanism for invading and pinching off portions of the cell membrane, allowing the entry of such molecules as MVs.^104^ MVs from *Helicobacter pylori*,^107^ *Lactiplantibacillus plantarum* BGAN8, and nonpathogenic *E.coli* Nissle 1917 (*Ec*N) and ECOR12^108^ are taken up by host cells via this mechanism.

Regions of the plasma membrane enriched in sphingolipids, and cholesterol are known as lipid-rafts. The clustering of cholesterol (which is the major component of the lipid raft) and other lipids in these domains allows the curvature of the membrane, driving the formation of invaginations in the host cell and entry of particles such as MVs into the cell.^104^ MV cargos also aid in facilitating entry into host cells via lipid raft-mediated processes. MVs from *Pseudomonas aeruginosa*^104^ and *Moraxella catarrhalis*^68^ have been shown to be taken up by the host cell via lipid raft machinery. Caveolin-mediated endocytosis involves the presence and the oligomerization of caveolin in lipid rafts which give rise to the formation of caveolae – cave‐shaped invaginations that are around 80 nm in diameter and are formed on the cell membrane, with cholesterol, caveolins, and sphingolipids in abundance.^104,109^ Just as in CME, dynamin is also required here.^109^ MVs from *V*. *cholerae*^110^ and *Haemophilus influenzae*^111^ have been demonstrated to enter the host cell via this mechanism.

Lastly, direct membrane fusion has been demonstrated as another mechanism of MVs’ entry into host cells.^104^ Membrane fusion preferentially take-place at lipid-raft regions and many studies have reported an increased surface area of the host membrane upon the addition of MVs-membrane on a model membrane with dye-labeling procedure.^104,112^ MVs from *P*. *aeruginosa*^113^ and *Legionella pneumophila*^112^ were taken up via this mechanism. On interaction with eukaryotic cells, the cargo(s) is/are delivered to the host, and appropriate function(s) mediated.^64,68^

#### 2.3.6. Immune modulation

MVs can influence host immune responses by modulating the expression of immune-related genes and by directly interacting with immune cells. They contain immunomodulatory molecules that can target host innate immune pattern recognition receptors (PRR) such as Toll-like receptors (TLRs) and Nod-like receptors (NLRs) signaling pathways, thereby stimulating the release of pro-inflammatory cytokines and chemokines, which attract immune cells to the site of inflammation.^114^ As a result of the small sizes of MVs and their immunogenicity, their interaction with innate immune cells (macrophages and neutrophils), antigen-presenting cells^115^(dendritic cells), and/or adaptive immune cells (T- and B- cells), leads to the generation of various immune responses as illustrated in Figure 5.^115,116^ For instance, the detection of LPS and LOS by TLR-4 results in the activation of nuclear factor-kappa B (NF-κB) and the release of proinflammatory cytokines. MVs of many pathogenic Gram-negative bacteria, including *E*. *coli* and *P*. *aeruginosa* can activate TLR-4.^117,118^ Additionally, MVs of Gram-positive bacteria such as *S. aureus* contain lipoproteins and other components that activate TLR-2 signaling in epithelial cells and macrophages, eliciting pro-inflammatory cytokine responses.^57,59,119^ Internalized MVs can also activate host cytosolic PRRs. Almost all peptidoglycans from Gram-negative bacteria have a conserved structural motif that is recognized by NOD1. Entry of MV-associated peptidoglycan into epithelial cells activates NOD1, leading to the activation of NF-κB and the upregulation of human β-defensins 2 and 3.^120^ NOD2, which detects a conserved peptidoglycan motif exclusive to both Gram-positive and Gram-negative bacteria, is also activated.^119,121^ Nucleic acids contained in MVs also activate NOD2, resulting in NF-κB activation, as seen in *S*. *aureus*-derived MVs.^119^ MVs from the probiotic *Ec*N and the commensal ECOR12 indirectly activate the innate immune response in IECs. These MVs activated NOD1 signaling pathways in IECs and subsequently triggered NF-κB signaling through the NOD1-RIP2 pathway.^108^ Another study reported that MVs from *Ec*N directly activated DCs, and these activated DCs induced the differentiation of Treg cells (FOXP3+).^122^ These studies show that MVs are effective in modulating intestinal immune responses and can be strategically applied as novel therapeutic agents in IBD.

Additionally, MVs have been implicated in bacterial pathogenesis as they can serve as long-distance delivery vehicles to stimulate the immune system, promote host colonization, and enhance immune evasion. Certain immunogenic molecules such as flagellin, peptidoglycan, toxins, and LPS, which stimulate the host immune system through TLRs^123^ and/or NLRs^108^ are contained in MVs. These molecules are also linked to some virulence factors of the bacteria, including adherence, invasion, immune system modulation, and antimicrobial resistance. MVs can also contain more than one virulence factor simultaneously.^20,47^ Detailed exploration of immune modulation by MVs with respect to IBD is found in succeeding sections.

#### 2.3.7. Microbiota homeostasis

Studies have shown that MVs are intimately involved in the communication between the gut microbiota and the host via a complex network of signaling pathways. These MVs play critical roles in the modulation of the gut microbiota homeostasis as they shape the immune responses of the host. MVs from *Clostridium butyricum*,^124^ *Lactobacillus rhamnosus*,^31^ *Lactobacillus plantarum*,^30^ *Akkermansia muciniphila*,^29^ *Faecalibacterium prausnitzii*,^125^ among others have been reported to efficiently modulate the gut microbiota balance via various mechanisms which are further explored in subsequent sections. MVs from many pathogenic bacteria such as *Fusobacterium nucleatum* (*Fn*) and *E*. *coli* can contribute to dysbiosis of the gut causing an imbalance in the gut microbiota homeostasis.

## 3. MVs and their potential role in IBD

### 3.1. MVs and the gut-microbiota

Crosstalk between epithelial and immune cells is crucial for maintaining intestinal homeostasis in the human gut. MVs secreted by intestinal bacteria can diffuse in the intestinal microenvironment or enter the bloodstream. After the internalization and cargo delivery of MVs into their target cells, specific signaling pathways for further processes are activated.^126^ It is interesting to note that MVs produced by a species of bacteria can impact the growth, reproduction, and colonization of members of the producing species differently. The MVs in the gut can be beneficial or harmful to the microflora and the host cell. In the host, for instance, MVs can regulate immunity (via interactions between epithelial and host cells) and promote the growth and colonization of probiotics, thereby maintaining microbial homeostasis. These are favorable to the host. MVs produced by commensal and probiotic bacteria in the human GIT can facilitate interactions amongst the host’s epithelial and immune cells, maintain microbiota homeostasis, and offer protection against diseases.^97,127^ On the other hand, MVs from pathogenic bacteria can damage the host’s mucosal barrier, causing harmful inflammatory storms to the host.^128^

Dysbiosis, an imbalance in the gut microbiota, plays a crucial role in the onset and progression of IBD. This imbalance contributes to the development of IBD through various mechanisms, including changes in the production and release of MVs. An important characteristic of IBD is a shift in the composition of the gut microbiota, typified by a decrease in beneficial bacteria and an increase in harmful bacteria.^30,31,129^ This dysbiotic state of the gut leads to alterations in the production and release of MVs, which results in the inhibition of colonization by probiotics and an increase in the growth and colonization of gut pathogens, culminating in inflammatory processes, a marked symptom of IBD.

### 3.2. MVs in the pathogenesis of IBD

As summarized in Table 2, several lines of evidence suggest that MVs play a crucial role in the development and progression of IBD, a chronic inflammatory condition affecting the gastrointestinal tract.

**Table 2.** Bacterial MVs in the pathogenesis of IBD.

S/N	MVs Origin	Impact in host	Model	
1	*B. thetaiotamicron*	Fulminant colitis in *dnKO* mice	*In vivo*	^130^
2	ETEC	Strong proinflammatory activity	*In vitro*	^131^
3	EHEC	Strong proinflammatory activity	*In vitro*	^132^
4	AIEC	Strong invasive ability	*In vitro*	^133^
5	*E. coli* BL21	Promotes recruitment of caspase‐5 and PIKfyve to early endosomal membranes via SNX10 ultimately resulting in intestinal barrier dysfunction	*In vitro* and *In vivo*	^71^
6	*F. nucleatum*	Reduced the levels of ZO-1, Claudin-1 and occludin, MUC1 and 2, polarized macrophages to M1 phenotype dysregulating the epithelial barrier integrity; Increased secretion of IL-8, TNF-α, IL-1β, IL-6, and iNOS, downregulation of IL-10	*In vivo*	^27,28,134^
7	*F. tularensis*	Facilitates the entry of the bacteria into host cells, promoting bacterial colonization	*In vitro*	^135^

EHEC – Enterohemorragic *Escherichia coli*, ETEC – Enterotoxigenic *Escherichia coli*, AIEC – Adherent invasive *Escherichia coli*.

#### 3.2.1. MV-induced disruption of intestinal epithelial barrier integrity

**Figure 6.**
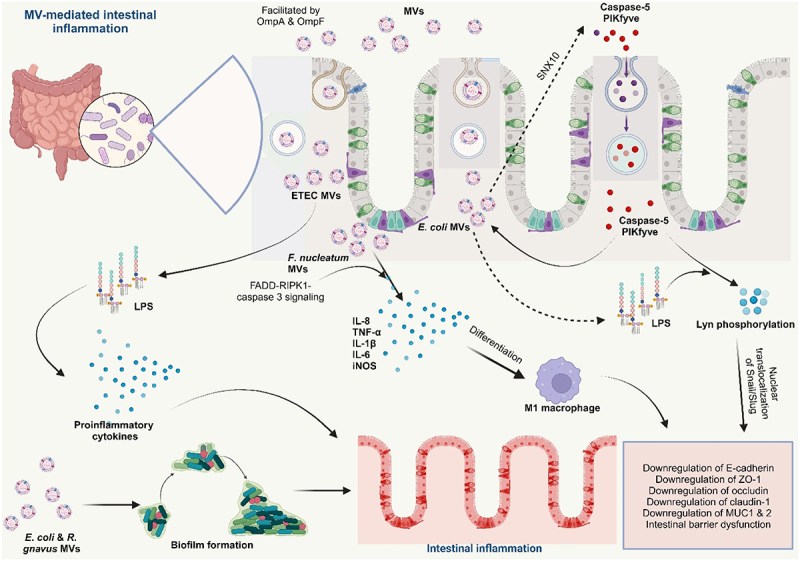
Bacterial membrane vesicles in the pathogenesis of IBD.

MVs from certain pathogenic bacteria in the GIT can lead to intestinal barrier dysfunction, a major symptom of IBD. MVs can disrupt the integrity of the intestinal epithelial barrier, allowing bacteria and their products to translocate into the lamina propria, the layer of connective tissue beneath the epithelium. This translocation further stimulates the immune system and contributes to chronic inflammation. Internalization of MVs from *E. coli* BL21 by intestinal epithelial cells occasions a cascade that involves sorting nexin 10 (SNX10) and LPS release from the MVs into the cytosol. The presence of cytosolic LPS leads to further downstream processing that culminates in intestinal barrier dysfunction, promoting inflammation in the gut.^71^ *Fn*-MVs significantly reduced the levels of tight junction proteins ZO-1, claudin-1, and occludin, as well as MUC-1 and −2, dysregulating the epithelial barrier integrity in colitis mice.^28^ Another study reported that *Fn*-derived MVs downregulated tight junction proteins ZO-1 and occludin, resulting in epithelial barrier dysfunction both *in vitro* and *in vivo*. The exacerbation of colitis by the MVs was linked with *Fn*-MVs facilitated downregulation of miR-574-5p expression and activation of autophagy^134^ (Figure 6).

Membrane vesicles (MVs) from pathogenic bacteria promote inflammation in the gut. MVs from ETEC, after internalization by intestinal epithelial cells, release their LPS, inducing the release of strong proinflammatory cytokines. MVs from *E*. *coli* BL21 promote the recruitment of caspase-5 and PIKfyve upon internalization by intestinal epithelial cells, also resulting in the release of their LPS into the cytosol, which culminates in intestinal barrier dysfunction. *Fn*-MVs triggered an upregulation of the proinflammatory cytokines IL-1β, IL-6, TNF-α, and iNOS and downregulation of anti-inflammatory IL-10 *in vitro* and *in vivo*. These MVs also enhanced apoptosis of intestinal epithelial cells by inducing the pro-inflammatory M1 phenotype, resulting in intestinal barrier dysfunction via FADD-RIPK1-caspase 3 signaling. They significantly reduced the levels of tight junction proteins ZO-1, claudin-1, and occludin, as well as MUC-1 and −2, dysregulating the epithelial barrier integrity. MVs from *E. coli* and *Ruminococcus gnavus* have been found to increase biofilm formation in the gut, limiting the efficacy of host defense factors and antibiotics against the parent bacterium.

#### 3.2.2. MV-induced modulation of host immune responses and delivery of virulence factors

MVs can influence host immune responses in IBD by modulating the expression of immune-related genes and/or by directly interacting with immune cells. These interactions can lead to an imbalance in immune responses, contributing to the chronic inflammatory characteristic of IBD. MVs from IBD-associated bacteria contain pro-inflammatory molecules, such as LPS and flagellin, which can activate TLRs on host cells. TLR activation triggers inflammatory signaling pathways that lead to the production of pro-inflammatory cytokines, contributing to the inflammatory state of IBD.

Interaction of LPS from bacterial-associated MVs with PBMCs resulted in the strong production of proinflammatory cytokines IL-6, IL-8, MCP-1, and MIP-1α.^136^ MVs from *Fn* promoted the secretion of proinflammatory cytokines IL-8 and TNF-α *in vitro* in colonic epithelial cells.^27^ *Fn*-MVs triggered an upregulation of the proinflammatory cytokines IL-1β, IL-6, TNF-α, and iNOS^28,134^ and downregulation of anti-inflammatory IL-10 *in vitro* in intestinal epithelial cells and *in vivo* in colitis mice.^134^ Increased levels of F4/80+ iNOS+M1-like macrophages were also reported; thus, *Fn*-MVs enhanced apoptosis of intestinal epithelial cells *in vivo* by inducing the pro-inflammatory M1 phenotype resulting in intestinal barrier dysfunction in UC (Figure 6). The FADD-RIPK1-caspase 3 signaling mediated this action of *Fn*-MVs and serves as a basis for further studies.^28^ In their study, Tulkens and colleagues also demonstrated a significant increase in the bacterial MVs associated with LPS activity in patients with intestinal barrier dysfunction such as IBD.^136^

A study by Durant and fellow researchers, found the possibility that the presence of IBD could most likely affect the responses of immune cells to otherwise beneficial MVs from commensal bacteria. In their study, they demonstrated that DCs are important APCs that can produce and respond to IL-10 to regulate immune responses and microbial tolerance. However, DC subsets are altered in IBD, and a decline in the numbers of CD103+ DCs in the colon of both UC and CD patients compared to healthy controls was reported, supporting a loss of regulatory DCs in IBD.^137^ Compared to healthy controls, *Bt*-MVs were unable to induce the expression of IL-10 in colonic DCs of UC patients and elicited a significantly lower proportion of DCs that expressed IL-10 in the blood of both CD and UC patients.^137^

The localization of *Bt*-associated antigens to host immune cells (macrophages) through the MVs of *Bt* with sulfatase activity was shown to be the primary cause of the fulminant colitis observed in genetically-susceptible *dnKO* mice treated with the bacterium. However, upon deletion of the anaerobic sulfatase maturating enzyme (anSME) from the bacterium, its ability to stimulate colitis in *dnKO* mice was remarkably abolished. This would mean that access of *Bt*-MVs to host immune cells was sulfatase-dependent and that the MVs of this bacterium and associated enzymes promote inflammatory immune stimulation in genetically susceptible hosts.^130^ The colonic macrophages of *dnKO* mice gavaged with wild-type, WT-*Bt* revealed a significant upregulation in the levels of pro-inflammatory markers COX-2, TNF-α, and IL-1β as compared to mice treated with PBS and ΔanSME *Bt*.

Many pathogenic *E*. *coli* have been reported to significantly promote the progression of IBD. The association between adherent invasive *E*. *coli* (AIEC) and IBD progression has been reviewed by several studies,^138–140^ AIEC strain LF82 recovered from a chronic lesion of a CD patient demonstrated great invasive ability in intestinal epithelial cells *in vitro*. The outer membrane proteins, OmpA and OmpC found in their MVs were identified as the virulence factors responsible for their invasiveness.^133^ Moreover, some other harmful *E*. *coli* associated with IBD, enterotoxigenic *E. coli* (ETEC) and enterohemorrhagic *E. coli* (EHEC)^141^ release MVs containing toxins (such as EHEC cytolysin ClyA and cytolethal distending toxin V, ETEC heat-labile enterotoxin (LT)) that can damage host cells, exacerbate inflammation, promote bacterial colonization, and consequently, disease progression.^104,141^ The strong induction of the proinflammatory cytokine IL-8 by ETEC MVs was also reported to occur via the internalization of their MVs and subsequent delivery of their LPS (contained in the MVs) to intestinal epithelial cells, which is then recognized by novel caspase- and RIPK2-dependent pathways.^131^ Underacylated LPS-derived ETEC OMVs showed similar uptake dynamics but less proinflammatory potency than MVs derived from WT ETEC, suggesting that this identification is likely due to the detection of the lipid A moiety.

#### 3.2.3. Promotion of bacterial colonization

MVs play significant roles in IBD development and progression by facilitating the colonization of harmful bacteria in the gut of IBD patients. They can achieve this by the various mechanisms described below.

Some MVs contain adherence factors that facilitate the entry of harmful bacteria into host cells, increasing their chances of colonizing the gut. The MVs of *Francisella tularensis* were reported to be involved in the entry of the bacteria into macrophages.^135^ Furthermore, dysbiosis enables the overgrowth of harmful bacteria in the gut, resulting in the increased secretion of their MVs in the gut lumen. Studies have also shown that IBD patients exhibited elevated levels of MVs in their feces compared to healthy individuals,^142^ which has been attributed to the dysbiotic gut microbiota associated with IBD. As reported above, MVs from pathogenic *E*. *coli* release toxins that damage the host cells facilitating their colonization in host cells.^104,141^ Again, the disruption of the integrity of the intestinal epithelial barrier by MVs allows the influx of harmful bacteria into the lamina propria, the layer of connective tissue beneath the epithelium. This translocation provides harmful bacteria access to nutrients and a protected environment for colonization.^28,143^

Another mechanism is via biofilm formation – a community of harmful bacteria embedded in a matrix of extracellular polymeric substances (EPS). Biofilms provide a protective environment for bacteria, making them more difficult for the immune system to eliminate. Additionally, biofilms release toxins and other inflammatory mediators that can contribute to the chronic inflammatory characteristic of IBD.^144^ MVs have the capacity to protect biofilms from host immune attack and antimicrobial agents, thereby promoting their persistence in the gut^145^ (Figure 6). Many pathogenic MVs-producing microorganisms such as enterotoxigenic *Bacteroides fragilis*, *E*. *coli*, *Ruminococcus gnavus* among others, which are known to be increased in the dysbiotic gut of IBD patients, have been found to form biofilms in the ileum and right-sided colon of the gut.^146,147^ Additionally, as MVs interact with host cells and modulate their signaling pathways, they can suppress immune responses, ultimately creating a more favorable environment for colonization by harmful bacteria.^28,134^

## 4. Bacterial MVs as diagnostic biomarkers of IBD

MVs can be analyzed for their content of specific molecules or signatures that could serve as biomarkers for IBD diagnosis and disease monitoring. Metagenomic profiling of patients with CD (active and remission) showed that the microbial community structure of stool-derived MVs was significantly different from the stool-derived microbiome in relation to healthy controls. Consequently, 16S rRNA sequencing of fecal-derived MVs was reported as more suitable as a diagnostic biomarker for IBD than just 16S rRNA sequencing of the bacterial population in the feces of IBD patients.^148^ Another metagenomic profiling study corroborated the above. Heo et al.. (2023) revealed that the analysis of gut microbe-derived MVs was more effective than stool microbiome analysis at differentiating patients with IBD from healthy controls.^149^ Yet, Kang reported that even though colitis induction resulted in a change in the gut-bacterial composition, a more drastic change was observed in the composition of bacterial-derived fecal MVs. Metagenomics of MVs composition in stool samples of dextran sulfate sodium (DSS)-induced colitis in mice revealed a decrease in the MVs of *Akk* and *Bacteroides acidifaciens* and an increase in MVs from TM7 phylum, particularly in DQ777900_s and AJ400239_s species.^150^

While several miRNAs are associated with disease origin and development, some have been found to be pathology-specific.^151^ Accumulating evidence reveals that significant levels of miR-21, miR-155, and miR-223 presented by IBD patients could be potential biomarkers for IBD.^152^ As a result, changes in miRNA expression profiles have been addressed for applications in the classification of early detection, prognosis, and diagnosis of IBD.^152^ The 2015 study by Polytarchou et al. revealed the association of miR-214 with the progression of IBD and how reducing its expression slowed the development of colitis and colitis-associated cancer in mice.^153^ Interestingly, recent studies have also shown that MVs affect the expression of miRNAs in IBD. The downregulation or upregulation of certain miRNAs in the presence of MVs tends to monitor not only the progression of IBD but also the alleviation of inflammation. For instance, the downregulation of miR-574-5p expression by *Fn*-MVs^134^ and restoration of miR-199a-3p expression by *Cb*-MVs^154^ demonstrate that miRNAs are intricately associated with MV exposure in IBD cases. With this knowledge, some specific miRNAs could serve as both diagnostic and potential targets for IBD treatment.

## 5. Potential therapeutic applications of MVs in IBD

Several studies have shown strong evidence for the possible application of MVs for therapeutic purposes in IBD. The findings from these studies are discussed below and have also been summarized in Table 3.

**Table 3.** Application of MVs in IBD therapy.

S/N	Parent bacteria	Impact on host	Model	References
1	*L*. *casei* and *L*. *plantarum*	Improved transepithelial electric resistance; Reduction in IL-8 and TNF-α cytokine and significant stimulation of IL-10	*In vitro*	^155^
2	*L*. *kefirgranum* PRCC-1301	Inhibited the loss of tight junction proteins occludin, claudin-1, and ZO-1; inhibited NF-κB signaling pathway Reduced levels of IL-2, IL-8, and TNF-α	*In vivo*	^156^
3	*C*. *butyricum*	Enhanced the secretion of mucins (MUC1, 2, 3, and 4) and claudin 1, 3, and ZO-1; Positively remodeled the gut microbiota; reduced levels of IL-6 and TNF-α; polarized macrophages to M2 phenotype	*In vivo*	^124,;157^
4	*A. muciniphila*	Stimulation of ZO-1 and mucus, Reduced IL-6. Positive remodeling of the gut microbiota; Selective promotion of the growth of beneficial bacteria via membrane fusion; enhanced mucosal IgA secretion via activation of DCs and B-cells in the Peyer’s patches, enhancing intestinal immune barrier function	*In vivo* and *In vitro*	.^29^ .^150^
5	*E. coli* Nissle 1917	Improved epithelial barrier function Reduced IL-1β, TNF-α, and IL-17	*In vivo*	^158^
7	*L*. *paracasei*	Upregulation of endoplasmic reticulum (ER) stress-associated proteins Downregulation of proinflammatory cytokines, increased expression of anti-inflammatory cytokines	*In vitro* and *in vivo*	^159^
8	*L. rhamnosus* GG	Increased bacterial α-diversity and restored the taxonomic imbalance of gut microbiota; reduced expressions of TNF-α, IL-1β, IL-6, IL-2	*In vivo*	^31^
9	*L. plantarum* Q7	Increased bacterial α-diversity and restored the taxonomic imbalance of gut microbiota; Reduced expressions of TNF-α, IL-1β, IL-6, IL-2	*In vivo*	^30^
10	*L. plantarum*	Remodeling the gut microbiota and increased abundance of SCFAs in the colon; promoted polarization of macrophages to M2 phenotype	*In vivo*	^160^
11	*B. fragilis*	Reduced expression of TNF-α and IL-17 and increased secretion of IL-10. Stimulated the production of IL-10 from T-reg cells	*In vivo*	^161^
12	*P*. *freudenreichii*	Reduction in NF-_k_B activation and IL-8 expression	*In vitro*	^162^
13	*P. pentosaceus*	Suppressed Ag-specific humoral and cellular responses and promoted M2-like polarization and MDSC differentiation; Upregulation of IL-10	*In vitro*	^163^
14	*B. thetaiotamicron*	Upregulation of IL-10	*In vivo*	.^164^
15	*F*. *praustnitzii*	Upregulated the expressions of ZO-1, occludin, IL-10,	*In vivo*	^125^

### 5.1. Modulation of intestinal epithelial barrier integrity

The integrity of the intestinal epithelial layer protects against invading pathogens and toxins. The formation of tight junctions (TJs) between adjacent IECs is very crucial in the maintenance of epithelial barrier function. Disruption of this epithelial barrier enhances intestinal permeability, a key predisposing factor to allergy, inflammation, and other metabolic diseases.^165^ The gut bacteria have been shown to strengthen the epithelial layer, and interactions of the immune system^166^ and MVs from the gut microbiota are crucial in the modulation of epithelial barrier integrity. Administration of MVs derived from *Ec*N to DSS-treated mice significantly improved epithelial barrier function in these mice (Fábrega et al., 2017). MVs from *Lactobacillus kefirgranum* PRCC-1301 (PRCC-1301-MVs) significantly inhibited the loss of tight junction proteins occludin, claudin-1, and ZO-1 thereby limiting epithelial permeability in the colon tissues of DSS-colitis mice.^156^ MVs from *Clostridium butyricum*, *Cb*-MVs significantly upregulated the secretion of colonic mucus (MUC-2) and tight junction proteins (ZO-1) compared to DSS-colitis mice.^157^ A report of *Cb* from another study indicated that their MVs significantly enhanced the secretion of higher amounts of mucins (MUC-1, −2, −3, and −4) as well as tight junction proteins claudin-1, 3, and ZO-1, improving DSS-damaged epithelial barrier.^124^ MVs from fecal fermentation exposed to miR-200b-3p restored intestinal barrier function via upregulation of tight junction molecules, claudin-3 and colonic MUC-1, and MUC-4 in DSS-colitis mice.^167^ Regulation of microbial tryptophan metabolites by *Cb*-MVs enhanced intestinal barrier integrity and reduced inflammatory activities in colitis mice.^154^ MVs derived from *Akkermansia municiphila*, *Akk* maintain the integrity of the intestinal barrier by the stimulation of the expressions of tight junction molecules, ZO-1 and occludin, as well as mucus in the intestinal lumen of the colon by entering the intestinal epithelial cells.^29^ MVs from *F*. *prausnitzii*, *Fp* significantly upregulated tight junction molecules ZO-1 and occludin in DSS-treated mice, significantly improving the epithelial barrier integrity.^125^

**Figure 7.**
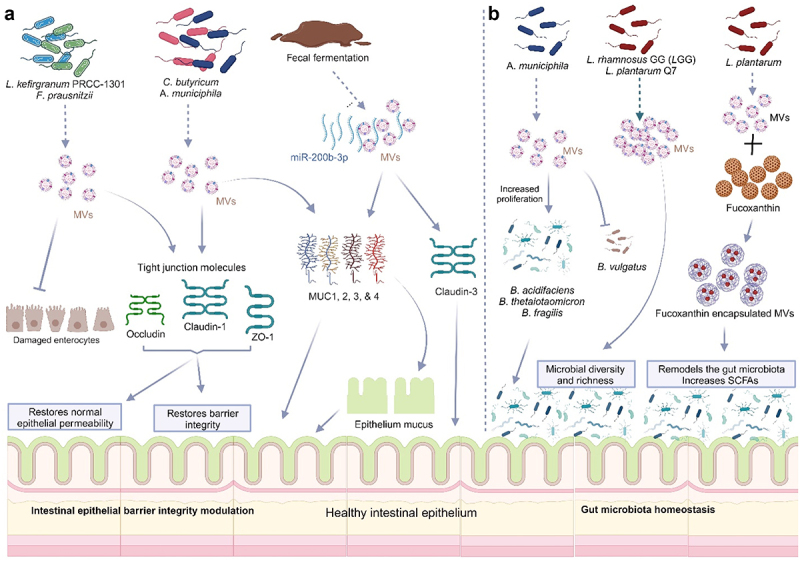
Bacterial membrane vesicles (MVs) repair the intestinal epithelial integrity and restore gut microbiota homeostasis.

These studies entail that MVs from probiotics have the capacity to repair the integrity of the intestinal epithelial barrier, subsequently eliminating the influx of bacteria and other agents into the lamina propria, thereby reducing inflammation and engendering IBD treatment (Figure 7).

(a) MVs from a variety of probiotics (*L. kefirgranum* PRCC-1301, *F. prausnitzii*, *C. butyricum*, *A. muciniphila*) have been implicated in the repair of damaged intestinal epithelial barrier resulting from colitis. They upregulate tight junction proteins occludin, claudin-1, ZO-1, and mucin 1, 2, 3, and 4. Exposure of MVs from fecal fermentation to miR-200b-3p also upregulated the intestinal epithelial mucins and claudin-3. (b) MVs from *A. muciniphila* selectively promoted the proliferation of beneficial bacteria *B*. *acidifaciens*, *B*. *thetaiotaomicron*, and *B*. *fragilis* by fusion but did not fuse with pathogenic *B. vulgatus* thereby inhibiting its growth. MVs from *L. plantarum* Q7, *L. rhamnosus* GG, and fucoxanthin-loaded MVs (FX-MVs) from *L. plantarum* re-modeled DSS-damaged gut microbiota promoting microbial diversity present and richness, grossly reducing the population of harmful bacteria and promoting the proliferation of probiotics and commensals. Increased short-chain fatty acids (SCFAs), were observed in FX-MVs re-modeled gut.

### 5.2. Restoration of gut microbiota homeostasis

Several studies have revealed that commensal and probiotic-derived MVs play fundamental roles in maintaining the stability of the intestinal microbiota. Not only do these MVs support the growth and colonization of beneficial microorganisms, but they also inhibit the growth and colonization of opportunistic and pathogenic microorganisms. The cargo delivered by MVs to the intestinal microflora, including enzymes, functional genes, and essential nutrients, enable them to thrive in the constantly changing microenvironment of the intestine.^128^ Although the intestines are host to a great diversity of bacteria, not all these bacteria have the capacity to produce MVs.

A recent study revealed that *Akk*-derived MVs restored the balance of the gut microbiota through membrane fusion by selectively promoting the proliferation of beneficial bacteria *B*. *acidifaciens*, *B*. *thetaiotaomicron*, and *B*. *fragilis*, by fusion but did not fuse with *B. vulgatus*, thus, had no growth benefit for it. This reveals the ineffectiveness of *Akk*-MVs in promoting the proliferation of potentially opportunistic *Bacteroides* species in DSS-induced gut disorder.^29^ MVs from *L. rhamnosus* GG (*L*GG)^31^ and *L. plantarum* Q7^30^ also ameliorated DSS-induced colitis and enhanced gut-microbiota balance by promoting the microbial diversity present. Oral gavage of *L*GG and Q7 MVs increased bacterial α-diversity and restored the taxonomic imbalance of gut microbiota induced by DSS. An increase in the number of Bifidobacteria and *Muribaculaceae* with a reduction in the Proteobacteria population was observed with oral administration of Q7-MVs, while *Helicobacter*, *Odoribacter*, *Desulfovibrio* were increased in DSS-treated mice, *Odoribacter*, *Alistipes*, *Muribaculaceae*, *Lachnospiraceae*_*NK4A136*_group, and *Akkermansia* were enriched in *L*GG-MVs treated mice. A greater abundance of *Odoribacter* was, however present in the *L*GG-MVs treated group compared to DSS-treated mice.^31^ *Cb*-MVs re-modeled the gut microbiota composition thereby improving DSS-induced colitis in mice.^157^ The relative abundances of *Lactobacillus*, *Bacteroidales*_*S24-7*_group, *Akkermansia* and *Bacteroides*, were significantly downregulated in response to DSS treatment, while MV treatment reversed these decreases.^157^ *Cb*-MVs also attenuated colitis in mice and modulated the gut microbiota by significantly reducing levels of pathogenic bacteria, including *Escherichia*/*Shigella*, and promoting a relative abundance of butyrate-producing *Clostridium sensu stricto*-1 and *Butyricicoccus*.^124,154^ Another study reported that MVs from normal feces of mice effectively reversed the composition of the intestinal microbiota, restored the intestinal barrier, and rescued colitis. Remarkably, MVs from fecal samples of colitis mice had similar effects after treatment with miR-200b-3pp.^167^ *Akk*-MVs treated group exhibited marked improvements in both richness and diversity of the gut microbiota compared to DSS-PBS-treated mice by promoting an increase in the relative abundances of several probiotic or commensal bacterial genera, including *Bacteroides*, *Lactobacillus*, and *Alistipes*, together with *Lachnospiraceae*_NK4A136_group and bacterium *f Lachnospiraceae*. The beneficial members of the *Bacteroides* genera in the MV-treated group were significantly upregulated. Additionally, *Akk*-MVs reduced the relative abundances of bacteria belonging to the phylum Proteobacteria, the largest phylum comprised of many pathogenic bacteria and regarded as a microbial signature of dysbiosis in the gut microbiota^29^ (Figure 7).

### 5.3. Immune system modulation

MVs from commensals and probiotic bacteria could elicit mucosal immunomodulatory responses by modulating the expression of immune-related genes and/or by directly interacting with immune cells in order to restore the immunological profile, alleviating colitis.

MVs derived from *L. paracasei*, reduced the activation of inflammation-associated proteins such as COX-2, iNOS, and NF-κB, as well as nitric oxide *in vitro*. Oral administration of these MVs *in vivo* offered protection against DSS-induced colitis. Upregulation of endoplasmic reticulum (ER) stress-associated proteins by these MVs was reported to be responsible for the anti-inflammatory effects observed.^159^ *Cb*-MVs restored the expression miR-199a-3p, which targets map3k4, thereby suppressing proinflammatory mitogen-activated protein kinase (MAPK) and NF-κB signaling pathways, ultimately contributing to *Cb*-MVs mediated anti-inflammatory effect.^154^ Pretreatment with *Akk*-derived MVs *in vitro* mitigated the production of the proinflammatory cytokine IL-6 from colonic epithelial cells upon stimulation by pathogenic *E. coli* MVs.^150^ Administration of MVs derived from *Ec*N to DSS-treated mice significantly reduced levels of proinflammatory cytokines IL-1β, TNF-α, and IL-17 in DSS-treated mice.^158^ Treatment of the macrophage cell line RAW 264.7 with *Ec*N-MVs improved the immune-related enzymatic and phagocytic activities of macrophages. Acid phosphatase which is associated with phagocytosis and clearance of exogenous substances by macrophages, was significantly improved upon stimulation with *Ec*N-MVs.^168^ Capsular Polysaccharide A (PSA) which is contained in *B. fragilis*-derived MVs has an immunomodulatory function and can prevent experimental colitis. Treatment of DCs with PSA-containing MVs prevented trinitrobenzene sulfonic acid (TNBS)-induced colitis in mice via suppression of the proinflammatory cytokines, TNF-α and IL-17, and increased secretion of IL-10. These MVs also enhanced the anti-inflammatory capacity of regulatory T-cells (CD4+CD25+Foxp3+T_regs_) and stimulated increased production of IL-10 from them. This study reported that the DCs’ action depends on Growth Arrest and DNA-Damage Inducible protein (Gadd45α) and that DCs recognize MV-associated PSA via TLR-2.^161^

There was a significant reduction in NF-κB activation and IL-8 expression in LPS-treated HT-29 human IECs upon pretreatment with *Propionibacterium freudenreichii*-derived MVs indicating their potent anti-inflammatory property, which partly depended on the activity of immunomodulatory proteins such as SlpB.^162^ MVs derived from *L. paracasei* inhibited LPS-induced proinflammatory cytokines and increased the expression of anti-inflammatory cytokines in HT-29 cells.^159^ PRCC-1301-MVs showed effective reduction in the levels of proinflammatory cytokines IL-2, IL-8, and TNF-α in DSS-treated Caco-2 cells as well as inhibition of the NF-κB signaling pathway in mice models of colitis.^156^ Kuhn and colleagues demonstrated that MVs from *L. casei* and *L. plantarum* strongly increased IL-10 anti-inflammatory cytokine. Another report also showed a significant reduction of TNF-α and increased IL-10 levels in macrophage inflammation models *in vitro* upon treatment with MVs from *L. plantarum* and *L. casei*.^169^ *Pediococcus pentosaceus*-derived MVs reportedly suppressed antigen-specific humoral and cellular responses and promoted M2-like macrophage polarization and myeloid-derived suppressor cell differentiation in bone marrow-derived macrophages and bone marrow progenitors, respectively, presumably in a TLR-2-dependent manner. Consistent with their immunomodulatory activity, MV-differentiated cells upregulated expressions of IL-10, arginase-1, and PD-L1 and suppressed the proliferation of activated T cells.^163^ *Cb*-derived MVs polarized macrophages to M2 phenotype^157^ and significantly reduced the levels of plasma LPS, IL-6, and TNF-α,^124^ ameliorating DSS-induced colitis in mice. MVs from fecal fermentation exposed to miR-200b-3p reduced levels of inflammatory markers IL-6, and TNF-α and increased the levels of IL-10 in DSS-induced colitis.^167^ *Bt*-MVs demonstrated upregulation of IL-10 production in colonic tissue and in splenocytes, ameliorating colitis in mice. Further interactions of *Bt-*MVs with the monocytic cell line THP-1 were shown to be mediated primarily by TLR-2.^164^

**Figure 8.**
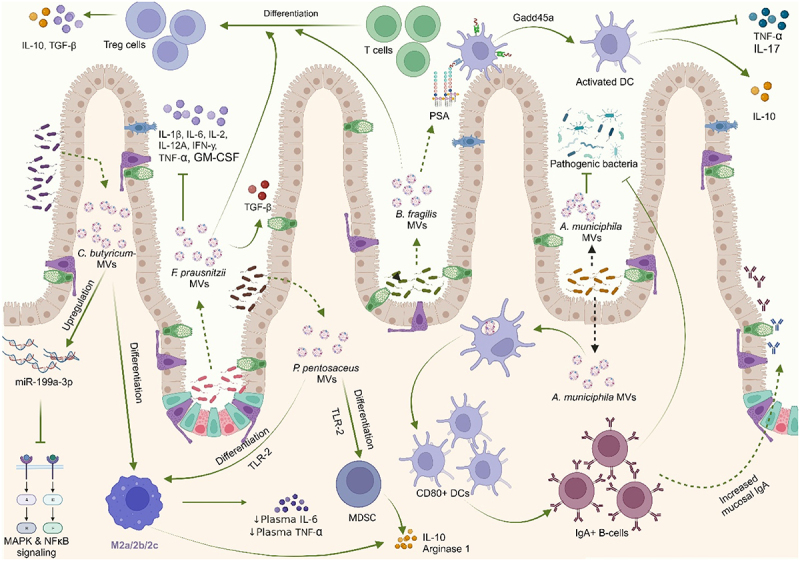
Bacterial membrane vesicles modulate the immune system under IBD conditions to enhance intestinal immune barrier function.

In their studies, Hao et. al. (2021) and Tong et. al. (2021) demonstrated that the increased genera in dysbiotic-colitis mice positively correlated with inflammatory cytokines. However, treatment with MVs from *Lp*-Q7 and *LGG* promoted the growth of anti-inflammatory bacteria genera, strongly alleviating colitis. They also reported that the expression of the pro-inflammatory cytokines TNF-α, IL-1β, IL-6, and IL-2 in these mice models of DSS-induced colitis were significantly downregulated by oral administration of the MVs.^30,31^ *Akk*-MVs elicited mucosal immunoglobulin A response by translocating into Peyer’s patches and then activating DCs and B cells, thereby enhancing the intestinal immune barrier function in order to prevent invasion by pathogens.^29^ *Fp*-MVs increased the ratio of T-reg cells in the colon tissue of colitis mice, downregulated the expression of the proinflammatory cytokines IL-1β, IL-2, IL-6, IL-12a, IFN-γ, TNF-α, and granulocyte-macrophage colony-stimulating factor (GM-CSF), and upregulated the anti-inflammatory cytokines IL-4, IL-10, and TGF-β in DSS-treated mice^125^ (Figure 8).

*C. butyricum-*MVs restored the expression miR-199a-3p, which targets map3k4, suppressing proinflammatory MAPK and NF-κB signaling pathways. These MVs also polarized macrophages to M2 phenotype and significantly reduced the levels of plasma LPS, IL-6, and TNF-α. *F. prausnitzii*-MVs increased the ratio of T-reg cells, downregulating the expression of proinflammatory cytokines and upregulating the anti-inflammatory cytokines. *A. muciniphila*-MVs elicited mucosal immunoglobulin A response by translocating into Peyer’s patches and then activating DCs and B-cells preventing invasion by pathogens. Interaction of DCs with capsular polysaccharide A-containing MVs of *B. fragilis* via Growth Arrest and DNA-Damage Inducible protein (Gadd45α) prevented colitis by suppression of TNF-α and IL-17 and increased secretion of IL-10. These MVs also stimulated increased production of IL-10 from T-reg cells. *P. pentosaceus*-derived MVs promoted M2-like macrophage polarization and myeloid-derived suppressor cell differentiation, eliciting increased expressions of IL-10 and arginase-1 from the differentiated cells.

### 5.4. Inhibition of MV release and interaction with their targets

Some studies have shown that certain agents could either prevent (block) the release of pathogenic MVs or inhibit their interaction with target cells/genes, mitigating inflammation in IBD patients. Wang and colleagues reported that deletion of sorting nexin 10 (SNX10) or treatment with its inhibitor DC-SX029 restored MV-induced intestinal barrier dysfunction and alleviated colitis in mice by blocking cytosolic MV-LPS release and further downstream signaling.^71^ The blockade of autophagy using chloroquine and inhibition of miR-574-5p/CARD3 axis ameliorated epithelial barrier dysfunction, autophagy activation, and subsequently, colitis severity mediated by *Fn*-MVs *in vitro* and *in vivo*.^134^ Hickey et al. reported that deletion of the anaerobic sulfatase maturating enzyme (anSME) from the wild-type *Bt* remarkably eliminated the ability of the MVs to stimulate colitis in *dnKO* mice. This revealed that access of *Bt*-MVs to host immune cells was sulfatase-dependent. The MVs of the bacteria and associated enzymes promote inflammatory immune stimulation in genetically susceptible hosts.^130^ The deletion of the yfgL gene in AIEC strain LF82 led to the release of fewer MVs by the bacteria and a gross reduction in their capacity to strongly invade intestinal epithelial cells.^133^

### 5.5. Genetically engineered MVs for targeted drug delivery

Due to MVs’ ability to penetrate physiological barriers that many synthetic delivery carriers cannot penetrate, they can finely serve as carriers of active components, such as anti-inflammatory drugs, or therapeutic nucleic acids.^160^ Moreso, the lipid bilayer of MVs offers stability and protection to the cargo, especially in the harsh environment of the GIT, leading to increased bioavailability of both the MVs and their encapsulated therapeutic agent. In addition, MVs can be engineered to display specific ligands on their surface, allowing for targeted drug delivery to sites of inflammation in the gut, thereby reducing off-target effects and improving efficacy. Unfortunately, their low secretion limits their widespread use, coupled with the lower yield of MVs loaded with active components.^160^

Liang and colleagues, however, successfully engineered MVs from the probiotic, *L*. *plantarum* on a large scale and even incorporated fucoxanthin (a dietary intervention for colitis) in these MVs. These FX-MVs gave a 150-fold yield and greater protein content compared with the naturally secreted MVs of the probiotic. Additionally, FX-MVs promoted the gastrointestinal stability of fucoxanthin and inhibited H_2_O_2_-induced oxidative damage by scavenging free radicals effectively, greatly ameliorating colitis.^160^ FX-MVs offered significant protection to colitis-mice, mitigating colonic inflammatory response. Interestingly, one mechanism by which FX-MVs attenuated colonic inflammatory response was by re-modeling the gut microbiota communities with a subsequent increase in the abundance of short-chain fatty acids in the colon (Figure 7).^160^ FX-MVs also promoted polarization of macrophages to M2 type and effectively suppressed levels of proinflammatory cytokines, improving colonic inflammation.^160^ Probiomimetics obtained from individually coupling MVs from *L*. *casei* and *L*. *plantarum* onto microparticles alleviated inflammation-induced loss of intestinal barrier function. They were reported to improve transepithelial electric resistance (an *in vitro* measure of barrier integrity function) caused by LPS-induced inflammation in Caco-2 monolayers, whereas native MVs could not.^155^ Probiomimetics also greatly ameliorated LPS-induced TNF-α secretion in colonic epithelial cells *in vitro* compared to native MVs. Reduction in IL-8 cytokine and significant stimulation of IL-10 secretion in these inflammatory environments were also observed, although *L. plantarum* MVs and *L. plantarum* MV-coated microparticles showed a higher anti-inflammatory effect than *L. casei* MVs and *L. casei* MV-coated microparticles.^155^

Nanoprobiotics, prepared from *Ec*N-1917 probiotic derived-MVs encapsulating manganese dioxide nanozymes demonstrated increased therapeutic ability of these MVs. These nanoprobiotics showed effective adherence to inflamed colonic epithelium and eliminated excess reactive oxygen species in the intestinal lumen of the murine IBD model. It is fascinating to note that these nanoprobiotics, in combination with the anti-inflammatory medicine, metformin, improved the overall richness and diversity of the gut microbiota, remodeled the pro-inflammatory state of the microenvironment, and displayed better therapeutic efficacy than commercially available IBD chemotherapeutics.^170^

MVs derived from *Bt* were engineered to express and stably deliver keratinocyte growth factor-2 (KGF-2), a human-derived therapeutic protein into the GIT of mice for protection against tissue inflammation and injury. These engineered *Bt*-MVs reduced disease severity and promoted epithelial repair and recovery in the DSS-induced colitis in mice.^97^

These studies illustrate that despite the challenges associated with MV secretion, their excellent plasticity allows for further manipulations for greater efficacy in IBD therapy.

### 5.6. MVs in IBD vaccines

Recent advances in immunological research present therapeutic vaccinations as an alternative in the treatment of many diseases. By stimulating the production of particular antibodies by the immune system, these vaccines may provide a means of treating IBD. Therapeutic vaccinations are a better option for managing IBD patients because of their safety and efficacy, as well as their ability to lessen the financial and healthcare burden associated with illness management. Studies on gut microbiota vaccines have upscaled in recent times. This entails using vaccines to induce the production of antibodies in the gut that target and act more specifically on the relevant pathogenic microorganisms – for the treatment of IBD.^171^ For instance, the involvement of different *E. coli* strains in the pathogenesis of IBD reveals that anti-*E. coli* vaccines could significantly mitigate intestinal inflammation. Daley and team found that a genetically attenuated ETEC vaccine, which was proven to be safe, improves *E. coli* flora dysbiosis by inducing a significant mucosal IgA response in the gut.^172^ Tran and colleagues also found that colitis-related characteristics such as inflammation, damage to epithelial cells, and constricted gut passageways improved in mice immunized with flagellin. This shows that chronic inflammatory illnesses could be treated with naturally occurring antibodies against flagellin or other pathogenic bacteria associated with IBD. Thus, stimulating the production of these particular antibodies with flagellin vaccination may be a useful strategy for treating intestinal inflammation in IBD patients. Furthermore, vaccines for IBD that target cytokines^171^ could also be formulated with an appropriate immune-stimulating bioparticle.

Although the intricate pathophysiology of IBD makes vaccination against a single pathogen insufficient to protect against the disease, MVs have several characteristics that make developing vaccines from them appealing. These include their capacity to display proteins from many sources, their inherent possession of pathogen-associated molecular patterns (PAMPs) that trigger potent immune responses, their nanoscale size for effective antigen processing and delivery, and their adaptability to be further altered, such as the combination of MVs with other nanomaterials that can help to improve vaccination efficacy by integrating the advantages of each individual component.^173^ MVs have been formulated into vaccines against viruses including human immunodeficiency virus, coronaviruses, human papilloma virus, hepatitis viruses, and influenza, and a wide variety of bacteria such as *Neisseria meningitidis*, *Neisseria gonorrhoeae*, *Acinetobacter baumannii*, *Streptococcus pneumoniae*, *S. aureus*, *Bordetella pertussis*, *Burkholderia mallei*, *Burkholderia pseudomallei*, *Edwardsiella tarda*, *E. coli*, *Klebsiella pneumoniae*, *P. aeruginosa*, and *Salmonella enterica*.^173^ CPS14^+^MVs vaccine prepared from the MVs of a probiotic *E. coli* strongly provoked an IgG class-switch combination with a Th1/Th2-balanced IgG subclass distribution without any adjuvant. This vaccine was also structurally stable with heat treatment. Mice of various ages showed broad efficacy for the CPS14^+^MV vaccination, and the humoral immune responses provoked by the vaccine remained in both the lungs and blood for a period of one year. The study revealed that the probiotic *E. coli* MVs-based vaccine platform provides a viable, broadly applicable defense against encapsulated pathogens.^174^ Although there are still debates on the possible involvement of *Mycobacterium avium* subspecies *paratuberculosis* (MAP) in the onset of CD, Aitken et al. successfully identified this organism in excised tissues of 18 IBD patients, with none detected in the 15 samples of non-IBD control.^175^ A recent, *in silico* vaccine design from MVs derived from MAP, showed that the multi-epitope vaccine obtained by stitching antigenic, immunogenic, and IFN-γ-inducing B-cell, MHC-I, and MHC-II epitopes through linkers could be a promising vaccine candidate against MAP, although both *in vitro* and *in vivo* experiments are required for solid confirmation.^176^

Although there are currently no direct studies on the development of MV-based IBD vaccines, these studies strongly show that the intrinsic characteristics of MVs and their ease of manipulation make their development and application in IBD vaccines feasible and imminent.

## 6. Challenges and future research directions

Despite the promising potential of MVs as therapeutic agents, several challenges need to be addressed before they can be widely used in clinical practice. These challenges include:

### 6.1. Development of efficient production process and administration of MVs in functional assays

Effective methods are needed to produce large quantities of MVs with consistent and desired characteristics. Many methods^69^ have been published for the extraction and purification of MVs, each presenting a range of advantages and disadvantages. For uniformity of MV studies across the globe and ease of comparative analysis, the best methods for the extraction and purification of MVs, which will also ensure they are produced in large quantities, need to be established.

Additionally, the study of Müller and colleagues revealed the significant effect of different culture conditions on the anti-inflammatory properties of vesicles derived from *L*. *casei* and *L*. *plantarum*.^169^ This entails that manipulation of vesicle-producing bacteria’s growth conditions could significantly affect the biological functions of these vesicles. As such, besides bioengineering, MVs from probiotic and commensal bacteria with proven anti-inflammatory activity could be manipulated in other ways to yield an even more excellent and efficient anti-inflammatory activity for the treatment of inflammatory-related diseases.

Many studies in MVs research quantify MVs for administration in functional assays using protein content characteristics only. The different protein assays (BCA, Lowry, Bradford, and Qubit assays) employed by Bitto and colleagues in their study showed significant variation in both the quantification and sensitivity of MVs produced by different species. Normalizing MVs by protein content lessened the ability to separate strain differences in the immunological functions of MVs.^82^ However, species-, strain-, and growth stage-dependent differences in MV cargo content were evident upon MV characterization by particle number. Performing immunological assays using an equivalent amount of MVs from *P*. *aeruginosa*, *H*. *pylori*, and *S*. *aureus* quantified based on their protein concentration masks the disparities in the amount of immunogenic cargo carried by MVs, and this impacts analyses of their immunostimulatory properties substantially. On the other hand, performing the same assays using an equivalent amount of MVs quantified by particle number revealed significant differences in their ability to be detected by PRRs, activate NF-κB, and induce an IL-8 proinflammatory response.^82^ Consequently, a standardized method for MV quantification in which a variety of factors that affect MV function, such as the bacterial growth conditions, growth stage, MVs extraction method, sample purity, MV size, particle number, and cargo content, are also reported, is strongly recommended.^82^ Biological comparisons of the functional differences between MVs across various bacterial genera, species, and strains will be made easier by this standardization. This will eventually produce consistency and comparability in the area of MV research.^82^

### 6.2. Deeper insights into the mechanisms of MV interactions with host cells

A deeper understanding of the complex interactions between MVs and host cells (intestinal epithelial cells and immune cells) is crucial for optimizing MV-based therapies.

### 6.3. Safety and efficacy evaluation

MVs are produced from either pathogenic bacteria, probiotics, or host commensals, and consequently, rigorous validations in the laboratory are still necessary for adequate evaluation of the appropriate dosage, safety, and efficacy of these MV-based therapies in IBD patients. These uncertainties hinder the translation of MV-based therapies from the laboratory to clinical trials, and specific studies that address them are urgently needed. Further studies targeted at MVs from particular bacteria types that have shown remarkable ability in the attenuation of colitis can be carried out to accurately ascertain these concerns, as it will make for quick and easy translation for clinical trials.

### 6.4. Targeting and delivery of MVs to specific sites

Mechanisms must be explored and enhanced to ensure that MVs reach the specific sites of action in the gut and deliver their therapeutic cargo effectively. It is also important that the MVs are stably delivered at adequate therapeutic quantities to their target sites. This is a major challenge, as it has been reported that some probiotic-derived MVs may face rapid clearance and possible dilution effects in the GIT, which may impair their therapeutic efficacy.^155^ The probiomimetics therapeutic system discussed earlier is quite advantageous and should be further explored since it limits the possibility of rapid clearance of the native MVs from the gut, leading to an increased concentration of MVs on the inflamed mucosal cells.^155^ Although the nanoprobiotics team reported an insignificant overt systemic toxicity in the treatment, it was overcome by the integration of cytokine storm calm with the biotherapy, ultimately culminating in the development of a safe and effective bionanoplatform for the effective treatment of inflammation-mediated intestinal diseases.^170^ Since the toxicity was successfully surmounted, it would be interesting to explore the potential inclusion of metformin in the engineered *Ec*N MVs. In simpler terms, incorporating the anti-inflammatory drug metformin into the engineered *Ec*N MVs could be a more potent alternative to administering each therapy on its own.

Therefore, future studies exploring these paths are encouraged in order to increase the bioavailability of MVs in the gut, thereby enhancing the therapeutic efficacy of these MVs.

### 6.5. The dual role of the commensal Bt-MVs in IBD

While some studies have reported that *Bt*-MVs can trigger the onset of colitis in a genetically susceptible host,^130^ or may not be effective in inducing the expression of IL-10 in both colonic and blood DCs of IBD patients,^137^ others have reported that the administration of *Bt*-MVs to DSS-colitis mice alleviated the symptoms of intestinal inflammation by upregulation of IL-10 production in colonic tissue and in splenocytes in mice.^164^ Further studies on this commensal bacterium and its MVs are required to determine the pathways and conditions that stimulate these various activities.

### 6.6. MVs-miRnas interaction

Several studies have shown that miRNAs are significantly altered in colitis, and interaction of MVs with some of these miRNAs can either promote or reverse colitis in mice via different mechanisms. For instance, *Cb*-MVs restored miR-199a-3p expression,^154^ treatment of MVs isolated from feces of colitis mice with miR-200b-3p rescued colitis in mice,^167^ *Fn*-MVs facilitated downregulation of miR-574-5p expression exacerbating colitis.^134^ These studies reveal that miRNAs could serve as potential targets for IBD diagnosis, progression, and therapy. Research focusing on determining the miRNAs implicated, mechanisms of action and interaction with MVs, and models for therapeutic applications are strongly advocated for.

### 6.7. MV-based IBD vaccines

The potential benefits of MV-based vaccines for IBD include effective immune response, targeted delivery, and improved safety. Future studies that focus on these benefits to develop vaccines from MVs against IBD are encouraged. Optimization of vaccine formulations, improving dosing regimens, and evaluating these vaccines’ long-term efficacy in preventing IBD flares and complications should be further considered.

## 7. Conclusion

Bacterial membrane vesicles have been described as major key players in the onset and progression of IBD, as well as in the treatment of the disease. Having adequate knowledge of the many factors that influence MV production and release is imperative for further studies in the area, particularly in the best approaches for manipulating MVs for the treatment of IBD. Many studies have reported that MVs from pathogenic bacteria induce strong pro-inflammatory responses that exacerbate inflammation, potentially resulting in IBD. Therapeutic agents that degrade these MVs in the gut lumen or block their release will be greatly needed to curtail these harmful effects. However, MVs from probiotics and some commensals have been shown to offer strong protection against the progression of IBD. It is therefore crucial that these MVs are further manipulated and effectively translated to different clinical trials of IBD treatment and management. Personalized therapy could even result from these since the makeup of the gut microbiota may show some slight uniqueness in each IBD patient. Lastly, there is also the possibility that MVs harbor specific molecules that could serve as biomarkers for IBD diagnosis and disease monitoring, enhancing their utility in IBD. The indispensable roles MVs play in IBD should be thoroughly considered, and a more profound insight into their mechanisms of action and interaction could become the next strategic area for notably reducing the epidemiology of IBD globally.
